# Damage integral and other predictive formulas for nonisothermal heating during laser exposure

**DOI:** 10.1117/1.JBO.27.3.035001

**Published:** 2022-03-31

**Authors:** Elharith M. Ahmed, Gary D. Noojin, Michael L. Denton

**Affiliations:** a SAIC, JBSA Fort Sam Houston, Texas, United States; b Air Force Research Laboratory, JBSA Fort Sam Houston, Texas, United States

**Keywords:** Arrhenius, photothermal, kinetics, damage integral, damage rate, microthermography

## Abstract

**Significance:** Physics-based models supply simulated temperature rises to photothermal damage rate models and provide comprehensive risk assessments for laser-induced damage. As the physics-based models continue to be refined, the damage rate models have not advanced. This peculiar lack of improvement is counterintuitive considering the damage integral (Ω), originally derived for isothermal heating events, and fails to accurately represent the nonisothermal heating from short laser exposures.

**Aim:** Derive a nonisothermal form of the damage integral and predict more accurately the damage induced by short laser exposures, as well as identify the role of heating rate in laser damage.

**Approach:** From first principles, we derived a version of the damage integral specific to the shape of thermal profiles rather than the square function provided by Arrhenius plots. We used previously published threshold thermal profiles, where all nonisothermal frequency factors ($A_{\mathrm{non}}$) solved all $\Omega_{\mathrm{non}}$ values to unity. Nonisothermal correction factors correct isothermal $A_{\mathrm{iso}}$ values.

**Results:** The $E_{a}$ values were identical for both the isothermal and nonisothermal conventions. Correction factor values for $\Omega_{\mathrm{iso}}$ ranged from 0.0 (20-s exposures at thermal steady state) to −0.93 (0.05-s exposures). Based on empirical results, we have derived a two-dimensional empirical formula that predicts the heating rate as a function of exposure duration and ambient temperature. Threshold peak temperatures ($T_{p}^{\mathrm{thr}}$) and threshold critical temperatures are mathematically determined without thermal profiles when appropriate $E_{a}$ and $A_{\mathrm{non}}$ values are established.

**Conclusions:** We have identified a modified damage integral that does not rely on the Arrhenius plot and provides a value for the frequency factor (A) that accounts for the nonisothermal nature of short laser exposures. The method, validated in our in vitro retinal model, requires thermal profiles recorded under threshold conditions, such as at minimum visible lesions or the boundary of cell death. The method is a new option for laser damage modelers.

## 1 Introduction

### 1.1 History of the Damage Integral (Ω)

In 1947, Henriques and Moritz published a series of systematic studies on the thermal damage kinetics of skin.^1^^–^^4^ The authors applied heated water or oil on the surface of human and porcine skin using a special manifold, and looked for redness over a wide range of temperatures (44°C to 60°C) and exposure durations (3 s to 5 h). Their damage results showed an exponential relationship to temperature and a linear correlation to time, indicating a first order reaction for the “temperature-time history.” Adopting the first order rate law of Arrhenius,^5^ which describes the kinetics of isothermal (constant temperature) chemical reactions, Henriques and Moritz determined their energy of activation ($E_{a}$) was similar in value to the energy of denaturation for most purified proteins. To provide a measure of accumulated thermal damage (Ω), Henriques integrated the Arrhenius rate law over time ($dt^{\prime }$), using the isothermal peak temperature ($T_{p}$) in kelvins, to produce the isothermal damage integral shown as

$$\Omega_{\mathrm{iso}}(\tau )=A_{\mathrm{iso}}\int_{0}^{\tau }e^{\frac{-E_{a}}{RT_{p}}}dt^{\prime },$$ (1)

where $A_{\mathrm{iso}}$ is the isothermal frequency factor ($s^{-1}$), $E_{a}$ is the activation energy ($J\;{\mathrm{mol}}^{-1}$), τ is the thermal exposure duration, and R is the universal gas constant ($8.314\;J\;{\mathrm{mol}}^{-1}\;K^{-1}$). With this mathematical representation of damage accumulation, Henriques defined an Ω value of 0.53 to represent reversible epidermal injury, and a value of 1.0 to represent complete necrosis at the basal epidermal layer.^4^ However, the authors noted that the relationship between temperature and time (exposure duration) skewed at the shortest exposures of a few min or less. It is important to note that the authors point out that it took 20 s for the basal epidermal layer to equilibrate thermally to the skin surface.^1^ This implies that for all exposure durations, the first 20 s displayed nonisothermal temperature rise, and then isothermal (thermal steady-state) conditions prevailed. At some value for exposure duration, the 20-s nonisothermal temperature rise will become negligible, and the entire exposure can then be approximated as isothermal. This description of early temperature ramp at the site of permanent damage (basal layer of epidermis) would explain the lack of continuity of the damage integral at shorter exposure durations.

The same issue came into play after the invention of the laser,^6^ when research on photothermal damage rate kinetics was accelerated. Laser exposure of absorbing tissue results in a rapid temperature rise that might seem instantaneous, but with proper temporal resolution, a ramp in temperature is evident. The nonisothermal nature of this initial photothermal temperature rise requires relatively long exposure durations to approximate an isothermal condition, just as was the case for Henriques and Moritz. Without an alternative, researchers in the field of laser–tissue interaction adopted the Arrhenius isothermal method described by Henriques decades earlier.

To obtain the Arrhenius rate parameters, A and $E_{a}$ for calculating Ω values, an accumulated damage level of unity (Ω value=1.0) is used to indicate the damaged state, and Eq. (1) can be linearized and rearranged to as

$$\mathrm{Ln}\;\tau =(\frac{E_{a}}{R})\frac{1}{T_{p}}-\mathrm{Ln}\;A_{\mathrm{iso}}.$$ (2)

**Fig. 1:**
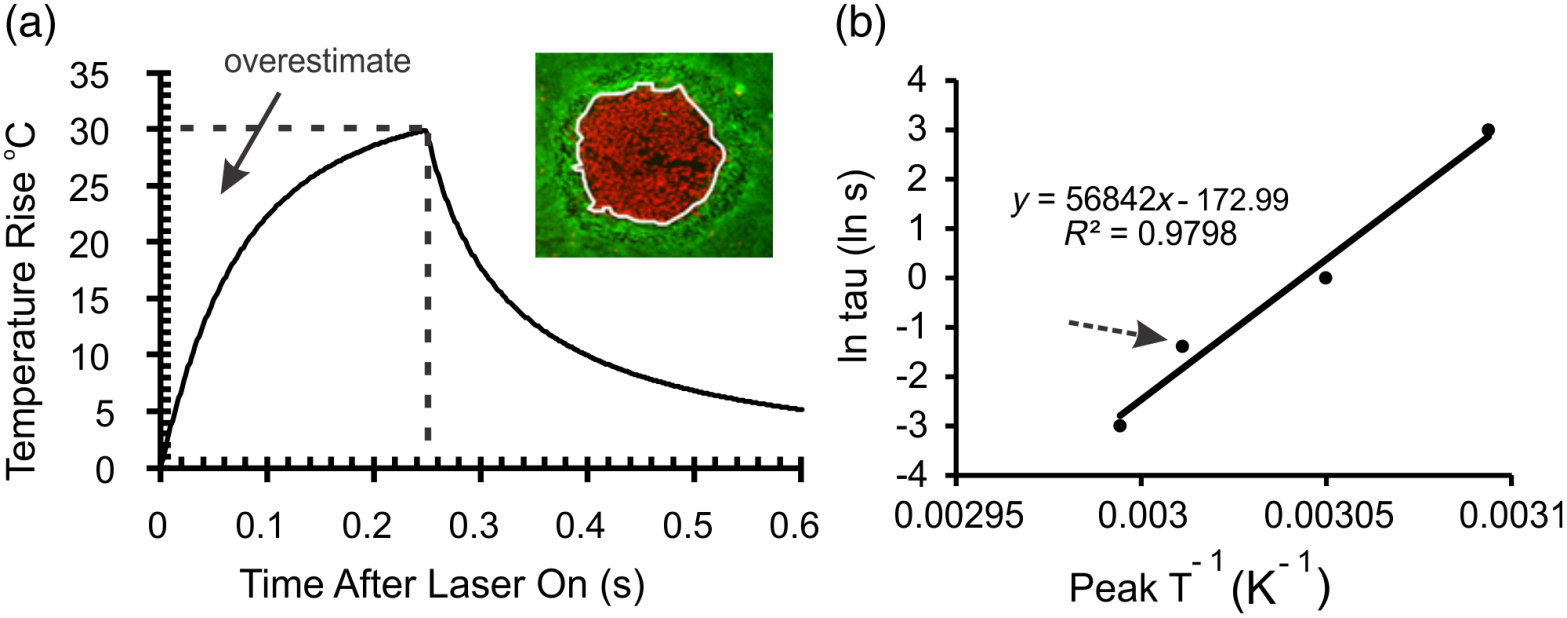
Comparing isothermal and nonisothermal heating. (a) Average threshold temperature-rise profiles for a 0.25-s laser exposure (2-μm at 30°C ambient temperature) showing the square function (dashed lines) used to determine the Arrhenius rate parameters (A and $E_{a}$). Arrow identifies region of overestimated temperature history used in typical Arrhenius method. Inset to (a) identifies the boundary of cell death, which is the source of the “threshold” thermal profile data. (b) Arrhenius plot to empirically determine $A_{\mathrm{iso}}$ (Exp[-y-intercept]) and $E_{a}$ (slope x R) using Eq. (2). Dashed arrow shows where the square function of panel (a) falls, as part of the average value on the plot of Eq. (2). Portions of (a) and all of (b) taken from Figs. 5(c) and 5(f) of Ref. 7, respectively. Other thermal profiles used in this study are provided in Fig. 2.

To illustrate the nonisothermal nature of short laser exposures we present, as reported in a previous paper,^7^ an averaged thermal profile (temperature versus time) for a 0.25-s exposure at 2  μm [Fig. 1(a)], and how that data were used to graph [Fig. 1(b)] Eq. (2). The dashed lines in Fig. 1(a) show the square function of temperature and exposure duration, and the arrow indicates the region of the isothermal assumption that causes an overestimate of the thermal dose delivered to the cells. The shape of the thermal profile dictates the degree of overestimation, and this area decreases as a percentage of the total area with extended exposure duration. From the straight line given by Eq. (2), the slope represents $\frac{E_{a}}{R}$ and the y-intercept gives $-\mathrm{Ln}\;A_{\mathrm{iso}}$ [Fig. 1(b)]. Figure 1(a) clearly shows why the Arrhenius integral, with constant temperature assumption, is suited for isothermal conditions, such as enzyme reactions where a small volume of enzyme is added to a reaction mixture that is already thermally equilibrated. Of course, the significance of this overestimation in photothermal damage can only be determined by devising an alternative approach that subtracts this overestimate, which is one purpose of this paper.

Six decades after the invention of the laser, no alternatives have been described that can match the damage prediction capability of the isothermal damage integral. Better methods for measuring and recording temperature responses of biological tissues are now available, such as thermal cameras with reasonable spatial and temporal resolution. Although advancing computational physics models that accurately predict temperature rises based on first principles^8^ is important, by focusing less on empirical measurements to support or refine the quality of damage prediction by the isothermal damage integral, there remains no alternative. Hence, no new data are available that address not only the isothermal, but several other known drawbacks^9^ of the damage integral, including the assumptions that the temperature and exposure duration dependence of the frequency factor are negligible. For a methodical evaluation of the significance of these drawbacks, one needs an experimental system that allows the collection of thermal data that reproducibly generates the same accumulated damage (Ω value), and a nonisothermal mathematical model that eliminates the overestimated portion of the thermal dose. The first requirement was described in a recent publication,^7^ and we will use that previous data to highlight our newly derived nonisothermal model.

To understand the significance of the data previously reported, it is important to understand the term minimum visible lesion, or MVL. The production of a photothermal MVL requires a minimal amount of laser energy to generate the threshold temperature rise leading to damage in a given cell or tissue type. However, due to differences in optical and thermal properties between similar samples, and even within the same sample, delivering the same “threshold” laser irradiance in repeated exposures will likely lead to three types of results; no damage, MVL damage, or damage that extended in size beyond the MVL stage. Thus, the term threshold laser dose, whether irradiance or radiant exposure, is less informative than a threshold temperature history. Only the MVL data are useful because of the desire to assign each exposure an Ω value of 1 for establishing relationships between laser exposure duration and ambient temperature ($T_{\mathrm{amb}}$). This would lead to inefficient data sets. Here, we denote the accumulated thermal history leading to a minimum visible lesion as a threshold thermal dose. A threshold thermal profile defines a threshold thermal dose, as experienced by the tissue during an MVL exposure or equivalent. The threshold thermal dose can be quantified, such as by the Arrhenius integral as a function of the thermal profile (Ω) to arrive at an accumulated damage value. An Ω value of unity (Ω=1) continues to be used routinely as the amount of accumulated damage barely (threshold) causing death.

### 1.2 Damage Rate Processes

Laser-induced thermal (photothermal) damage is a complex, multiphasic cellular response to increasing temperature over a specified time. The progression of damage involves thermal transitions (unfolding) of macromolecules, especially proteins, to the point of losing biological function. As the accumulation of nonfunctional biomolecules continues, the ability of the cell to repair the thermally induced stress diminishes until there is irreversible damage. As shown for whey proteins in solution, the rate of heating can influence the structure and solubility of proteins.^10^^,^^11^ Of particular interest is the notion that unfolded proteins have some chance of refolding, unless they begin to aggregate and become irreversibly “denatured.” The reversible protein unfolding process is considered a first-order reaction. deWit and Klarenbeek^10^ found that, although their whey protein began to unfold at 70°C for both heating rates of $1{}^{\circ}C\;\min^{-1}$ and $1{}^{\circ}C\;s^{-1}$, the temperature at which the protein started aggregating (also 60% unfolded in both groups) was drastically different. The slower heating rate produced 60% unfolded protein at 73°C, and the faster heating rate produced the 60% unfolded fraction at 85°C. Thus, for temperature rises, there are two kinetic rates (unfolding and aggregation) existing concurrently, depending upon both the peak temperature achieved and the rate of heating.

This example provides kinetics data from slow processes (0.17 to $1{}^{\circ}C\;s^{-1}$) relative to the rates of temperature rise produced by laser exposures. For instance, the exposure shown in Fig. 1(a) produced a heating rate of $120{}^{\circ}C\;s^{-1}$ (30°C rise in 0.25 s). Considering all the 2-μm laser heating data from Denton^7^ et al., the range of heating rate in their average threshold thermal profiles was from $7.5{}^{\circ}C\;s^{-1}$ (40°C ambient to 55°C peak in the first 2-s of a 20-s exposure) to $800{}^{\circ}C\;s^{-1}$ (20°C ambient to 60°C peak in 0.05 s). If we extrapolate the trend described by deWit^11^ above to the photothermal data, the amount of denatured protein would be low by the end of these short exposures. Perhaps cells are sensitive to a small amount of inactivated protein, especially a protein with a critical function. However, the extremely high protein concentrations within living cells relative to the test tubes of deWit would significantly increase the rate of aggregation of proteins once unfolded.^12^ This highlights that, although trends found in test tubes reveal important features for live cells, direct comparisons and interpretations require caution.

## 2 Methods

### 2.1 Derivation of Nonisothermal Damage Integral

Due to the complex physical changes in state and chemical aggregation leading to damage, approaching the kinetics as an overall composite system was an alternative starting point to the isothermal chemical reactions of Arrhenius.^5^ The currently accepted kinetic equation for a heterogeneous condensed phase system^13^ is

$$\frac{d\alpha }{dt}=f(\alpha )k(T).$$ (3)

The rate of conversion of the measured variable, α, was assumed to equal a separable mass-loss function, f(α), and a temperature dependent rate function, k(T).^13^^,^^14^ Think of α as the property of the material that was converted during the temperature dependent process. In laser bioeffects, this would be equivalent to the one or more proteins responsible for cellular damage when denatured. The degree of conversion of α (0.0 to 1.0) that causes cell damage can be arbitrary. Due to the complexity of the cellular components, including our lack of knowledge about the actual protein(s) involved in the damage rate process, α becomes impossible to measure. Therefore, assuming a separable f(α) and k(T) functions, the heterogeneous condensed phase system provides a good starting point. Considering the familiar Arrhenius rate equation to describe the temperature dependent rate function yields

$$k(T)=A_{\left(\tau \right)}e^{\frac{-E_{a}}{RT(t)}},$$ (4)

and substitution into Eq. (3) yields

$$\frac{d\alpha }{dt}=f(\alpha )A_{\left(\tau \right)}e^{\frac{-E_{a}}{RT(t)}}.$$ (5)

For variable heating rates, separation of variables and integration of Eq. (5) yields a measure of converted sample (g(α)):

$$g(\alpha )=\int_{0}^{\alpha }{\left[f(\alpha )\right]}^{-1}d\alpha =A_{\left(\tau \right)}\int_{0}^{\tau }e^{\frac{-E_{a}}{RT(t^{\prime })}}dt^{\prime }.$$ (6)

This formulation was an alternative derivation of the damage integral (Ω) [Eq. (1)] used by Henriques and Moritz and others.^4^^,^^7^^,^^15^^–^^21^ Classically, threshold thermal damage occurs when the integration in Eq. (6), with respect to α, generates a value of one at some time during a nonisothermal insult. The resulting equation is provided as

$$A_{\mathrm{non}}\int_{0}^{\tau }e^{\frac{-E_{a}}{RT(t^{\prime })}}dt^{\prime }={Ω}_{\mathrm{non}}(\tau )=1,$$ (7)

where $A_{\mathrm{non}}$ and $\Omega_{\mathrm{non}}$ represent the nonisothermal frequency factor and nonisothermal damage integral, respectively.

Operating Eq. (7) on thermal data shown in Fig. 1(a) entails the region below the line of the profile and represents the nonisothermal heating history. To quantify the region of overestimation shown in Fig. 1(a) (arrow), one can subtract the region under the profile from the region representing the square function (dashed box). To express the isothermal square function as Eq. (8), we replaced $T(t^{\prime })$ in the exponent of Eq. (7) with peak temperature ($T_{p}$), used the bulk time frame of τ, and set the equation equal to 1.0:

$$A_{\mathrm{iso}}e^{\frac{-E_{a}}{RT_{p}}}\cdot \tau =1.$$ (8)

We correct for these assumptions using an exposure duration dependent correction factor δc (τ), that also takes into account the variable heating rate. In essence, we define ${Ω}_{\mathrm{non}}(\tau )$ [Eq. (7)] as the false square function [Eq. (8)] minus a correction factor, δc (τ), as shown in Eq. (9):

$$A_{\mathrm{non}}\int_{0}^{\tau }e^{\frac{-E_{a}}{RT(t^{\prime })}}dt^{\prime }=A_{\mathrm{iso}}e^{\frac{-E_{a}}{RT_{p}}}\cdot \tau -\delta_{c}(\tau ).$$ (9)

However, researchers do not integrate the square function when calculating $\Omega_{\mathrm{iso}}$. Instead, modelers predict damage by integrating nonisothermal profiles using the $A_{\mathrm{iso}}$ obtained from the isothermal function [Eq. (2), Fig. 1(b)], as depicted in Eq. (10):

$$A_{\mathrm{non}}\int_{0}^{\tau }e^{\frac{-E_{a}}{RT(t^{\prime })}}dt^{\prime }=A_{\mathrm{iso}}\int_{0}^{\tau }e^{\frac{-E_{a}}{RT(t^{\prime })}}dt^{\prime }-\delta_{c}(\tau ).$$ (10)

Because of the fact that $E_{a}$ is the same value when solved via Eq. (2), rearrangement provides the simplified definition of $\delta_{c}(\tau )$

$$\delta_{c}(\tau )=\Omega_{\mathrm{iso}}-\Omega_{\mathrm{non}}.$$ (11)

Equation (11) indicates that the correction factor is applicable to the common usage of $A_{\mathrm{iso}}$ to determine the damage integral used by the laser research community ($\Omega_{\mathrm{iso}}$) and does not correct the entire step function [Eq. (8)]. As such, $\delta_{c}(\tau )$ is considerably smaller than that if modelers used Eq. (9). Clearly, the correction in Eq. (10) is entirely a function of the frequency factor correction, where $A_{\mathrm{iso}}$ is smaller than $A_{\mathrm{non}}$, which consistently leads to an underestimate of damage ($\Omega_{\mathrm{iso}}$ values $<\Omega_{\mathrm{non}}$) by the modelers. When using threshold thermal profiles, where the value for $\Omega_{\mathrm{non}}$ is defined as 1, the correction factor simplifies further to $\delta_{c}(\tau )=\Omega_{\mathrm{iso}}-1$, reinforcing the fact that the correction factor as defined in Eq. (10) is a negative value.

Upon additional rearrangements, we find the relationship between $\delta_{c}(\tau )$ and frequency factors, $A_{\mathrm{iso}}$ and $A_{\mathrm{non}}$ as

$$\delta_{c}(\tau )=\frac{\left(A_{\mathrm{iso}}-A_{\mathrm{non}}\right)}{A_{\mathrm{non}}},$$ (12)

and

$$A_{\mathrm{iso}}=A_{\mathrm{non}}(\delta_{c}(\tau )+1).$$ (13)

Substituting for $A_{\mathrm{iso}}$ in Eq. (2) yields

$$\mathrm{Ln}\;\tau =(\frac{E_{a}}{R})\frac{1}{T_{p}}-\mathrm{Ln}(A_{\mathrm{non}}(\delta_{c}(\tau )+1)),$$ (14)

which can be written as

$$\mathrm{Ln}\;\tau =(\frac{E_{a}}{R})\frac{1}{T_{p}}-(\mathrm{Ln}\;(\delta_{c}(\tau )+1)+\mathrm{Ln}\;A_{\mathrm{non}}),$$ (15)

where $T_{p}$ is the peak temperature to be maintained during the exposure to retain the same amount of accumulated thermal damage by the end of exposure. Having a y-intercept with two variables demonstrates that determining $A_{\mathrm{non}}$ and $\delta_{c}(\tau )$ from an Arrhenius plot is impossible. Hence, a different approach must be taken to determine $A_{\mathrm{non}}$ and $\delta_{c}(\tau )$.

### 2.2 Determination of $A_{\mathrm{non}}$ and $\delta_{c}(\tau )$ Using Individual Threshold Thermal Profiles

As a starting point for determining values for $\Omega_{\mathrm{non}}$, we use the simple rearrangement of Eq. (7) to yield

$$A_{\mathrm{non}}={\left[\int_{0}^{\tau }e^{\frac{-E_{a}}{RT(t^{\prime })}}dt^{\prime }\right]}^{-1},$$ (16)

where $T(t^{\prime })$ represents $\left[T_{\mathrm{amb}}+\Delta T(t^{\prime })\right]$. Given $E_{a}$, this method provides a value for $A_{\mathrm{non}}$ from each of our threshold thermal profiles. By means of the Arrhenius plot for each ambient temperature, $E_{a}$ values are determined from the slope. Using these ambient temperature-dependent $E_{a}$ values, we calculated mean $A_{\mathrm{non}}$ ± standard deviations for each set of exposure duration and ambient temperature using Eq. (16). Knowing the value for $A_{\mathrm{non}}$, we can use Eq. (12) to determine $\delta_{c}(\tau )$ for each threshold thermal profile as well, resulting in mean values of $\delta_{c}(\tau )$ ± standard deviations for each set of exposure duration and ambient temperature. Interestingly, one can calculate the $A_{\mathrm{iso}}$ value for each thermal profile by the incorrect assumption in Eq. (8) and rearranging it similar to Eq. (16). Therefore, with a relatively small, but significant number of newly generated threshold thermal profiles, one can assess the $\delta_{c}(\tau )$ [Eq. (13)] needed for correcting previously reported nonthreshold thermal profiles for a given sample type and laser system if desired.

### 2.3 Source of Threshold Thermal Profiles to Calibrate Nonisothermal Damage Integral

As described in the previous section, once an $E_{a}$ value for a given $T_{\mathrm{amb}}$ is known, individual thermal profiles can provide values for $A_{\mathrm{non}}$ using Eq. (16). In contrast to the traditional plotting of Eq. (2) over a series of laser exposure durations, the use of individual thermal profiles enabled the determination of mean $A_{\mathrm{non}}$ and $A_{\mathrm{iso}}$ values with standard deviations within the same exposure duration. Ideally, “threshold” thermal profiles would be used to determine the Arrhenius rate parameters because then, by definition, the damage integral would solve to a value of unity. We have postulated that, as long as the damage area was smaller than that of the laser footprint, cells at the boundary of damaged and nondamaged cells in exposed cultures should represent the MVL condition [white line in Fig. 1(a), inset].^7^ If this were true, it would open the door for generating threshold thermal profiles using a wide range of laser irradiances and damage areas. Damaged monolayers would provide MVL data without being limited to a small minimum lesion in the center of an exposed area.

Our method for determining the temperature history at the boundaries of damage, termed microthermography,^7^^,^^15^ uses high-speed and high-magnification thermal imaging, followed by an image overlay with postexposure fluorescence images indicating damaged areas. Spatially resolved temperature responses of nonpigmented retinal pigment epithelial (RPE) cells to 2-μm laser irradiation were recorded at 800 fps with an InSb-based camera (ThermoVision SC6000, FLIR Systems, Inc., Boston, Massachusetts), with an effective pixel pitch of about 8  μm at the image plane. Custom LabVIEW image processing routines performed the overlays with the appropriate stretching, resizing, and orientation changes. Once overlaid correctly, our software identified the thermal pixels associated with the boundary by defining a single-pixel region of interest (ROI) around the perimeter of the damaged area [white line in Fig. 1(a), inset]. The final extraction of the thermal history of the boundary ROIs also used the suite of LabVIEW programs. Large damage areas, no >90% of the laser area, provided a larger number of useful thermal pixels per boundary ROI, which strengthened the statistical power of the method.

**Fig. 2:**
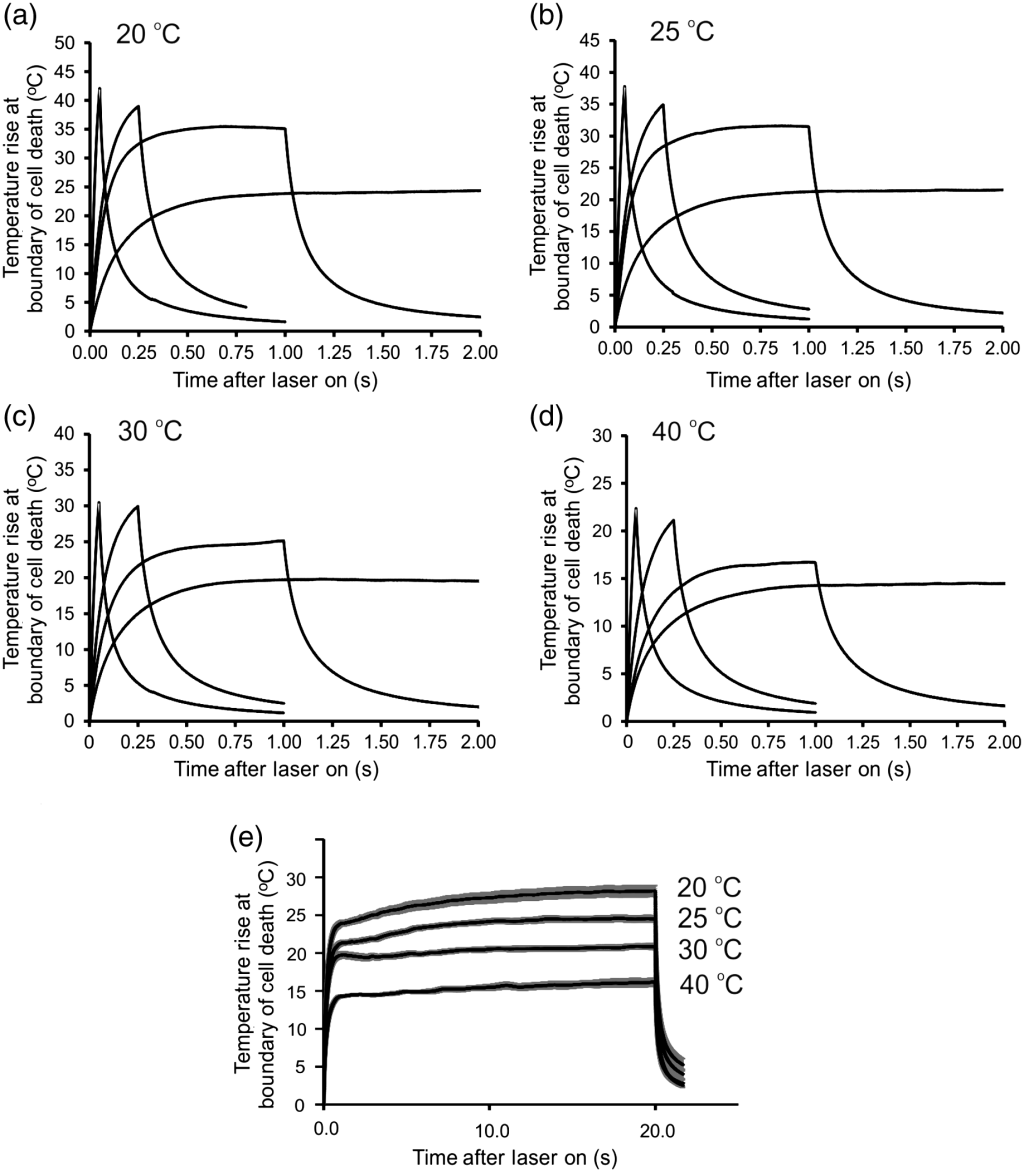
Threshold thermal profiles (temperature rises) for nonpigmented RPE cells exposed at 2  μm. Initial 2 s of thermal responses to 2-μm laser exposure at ambient temperatures of (a) 20°C, (b) 25°C, (c) 30°C, and (d) 40°C. Panel (e) provides full thermal profiles for the duration of the 20-s exposures at each of the four ambient temperatures. Temperatures, taken from the boundaries of cell death across a wide range of laser irradiances and damage sizes, are represented as the mean value (with SEM bars) at each time point. Taken from Ref. 7.

We chose 16 different sets of exposure parameters to investigate the relationship between threshold thermal dose of both laser exposure duration and the $T_{\mathrm{amb}}$ at which the cells were exposed. We investigated exposure durations of 0.05, 0.25, 1.0, and 20 s, which was much broader than our initial range of 0.1 to 1.0 s.^15^ Exposures at the four durations were delivered at four different ambient temperatures. The $T_{\mathrm{amb}}$ values chosen were 20°C, 25°C, 30°C, and 40°C. We ensured that the RPE cells were healthy throughout the duration (<15  min) of the experiments when held at these ambient temperatures. Multiple recordings (12 to 20) of thermal responses of cells in real-time with laser exposure provided the thermal data (Fig. 2 and Ref. 7) we used for each of the 16 different combinations of $T_{\mathrm{amb}}$ and τ.

In the prior paper, and using the typical isothermal method for determining $E_{a}$ and A, we verified that, within 10% experimental error, all 16 thermal profiles we extracted from the boundaries of cell death were equivalent to the same Ω value.^7^ Due to the threshold nature of the thermal profiles, we assumed an Ω value of 1.0 for each. Using empirically derived averaged values for A and $E_{a}$, each of the 16 average threshold thermal profiles were corrected by scaling the temperature values at each time point by a small increment, until the Ω value solved to 1.0. The scaling factors ranged from 0.6% to 10.3%. Therefore, the thermal profiles from our prior study are equivalent to those from an MVL exposure, allowing us to evaluate differences between isothermal and nonisothermal damage integrals in the current paper.

In preparing the 16 thermal profiles for use in this study, it became apparent that averaging the thermal profiles at each time point to generate one representative thermal profile, as shown in Fig. 2, was not optimum for data analysis. Here, we implemented the more rigorous strategy of a nearest-neighbor interpolation function, which determined numerical expressions of individual profiles. This generated only slight differences to values like $T_{p}$, critical temperature ($T_{\mathrm{crit}}$), and $E_{a}$ relative to the prior data, while providing superior statistical support to our new data. Here, we return to the fundamental aspects of the kinetic rate for a heterogeneous condensed phase system and derive a modified damage integral that removes the overestimated thermal dose. To highlight our results, we used the previously published thermal profiles to compare the differences in $E_{a}$ and A values associated with nonisothermal treatment relative to the traditional isothermal method. Again, the thermal profiles provided threshold temperature history because they were derived from the boundary of cell death [Fig. 1(a), inset]. As such, we will set the nonisothermal damage integral ($\Omega_{\mathrm{non}}$) to unity. The variance from unity found previously for the isothermal damage integral ($\Omega_{\mathrm{iso}}$) values provided a measure of error. Another chief outcome of the nonisothermal derivation is that once temperature and exposure duration dependent A and $E_{a}$ values are obtained for a given sample environment, one can predict threshold $T_{p}$ ($T_{p}^{\mathrm{thr}}$) and threshold $T_{\text{crit}}$ ($T_{\mathrm{crit}}^{\mathrm{thr}}$) without the need to integrate a thermal profile.

## 3 Results and Discussion

### 3.1 Traditional (Isothermal) Convention for Determining Arrhenius Parameters

Traditionally, determining the Arrhenius rate parameters, $A_{\mathrm{iso}}$ and $E_{a}$, using the Arrhenius plot [Fig. 1(b)] of Eq. (2) requires measuring peak temperatures for multiple laser exposure durations. This gives one value for each parameter across the entire range of τ values within a given ambient temperature. Table 1 provides the values for $A_{\mathrm{iso}}$ and $E_{a}$ that were determined with an Arrhenius plot using the interpolated thermal profiles from Ref. 7. The laser exposure duration (0.05±0.002, 0.250±0.002, 1.000±0.002, and 20±0.1  s) for each thermal profile was plotted versus the corresponding peak temperature [Eq. (2)]. The linearization of the data generated a single $A_{\mathrm{iso}}$ and $E_{a}$ parameter for each ambient temperature (Table 1). Both $A_{\mathrm{iso}}$ and $E_{a}$ values in Table 1 were greater than the values determined without interpolation.^7^ Differences due to interpolation had little effect (0.4% to 8.7%) on slope ($E_{a}$) values. However, due to slight changes in $T_{p}^{\mathrm{thr}}$ values, which propagated into slight changes in y-intercept values, there were large (three to five orders of magnitude) difference in the $A_{\mathrm{iso}}$ values between the prior and the current studies. These differences exemplify the large effect on $A_{\mathrm{iso}}$ with small changes in the y-intercept when taking the antilog function.

**Table 1:** Arrhenius rate coefficients from Arrhenius plot.

	From Arrhenius plot		
Ambient temperature (°C)	y-intercept	Arrhenius $A_{\mathrm{iso}}$ ($s^{-1}$)	Arrhenius $E_{a}$ ($J\;{\text{mole}}^{-1}$)
20.2±0.2	−146.19	$3.09\times 10^{63}$	399,161
24.8±0.2	−149.93	$1.30\times 10^{65}$	410,410
29.6±0.1	−189.88	$2.91\times 10^{82}$	518,706
40.0±0.1	−240.65	$3.26\times 10^{104}$	663,627

### 3.2 Nonisothermal Convention for Damage Integral

#### 3.2.1 Correction for nonisothermal damage integral is inherent in $A_{\mathrm{non}}$

The key to subtracting the overestimate in the isothermal square function (Figs. 1 and 2) is applying the nonisothermal correction factor, $\delta_{c}(\tau )$. However, calculating $\delta_{c}(\tau )$ using Eq. (12) requires that both $A_{\mathrm{non}}$ and the $E_{a}$ be predetermined. While the $E_{a}$ values generated by the Arrhenius plot (Table 1) represent both isothermal and nonisothermal heating [Eqs. (2) and (15)], the $A_{\mathrm{iso}}$ values represent the isothermal heating. Thermodynamically this makes sense because the threshold peak temperature represents the energy needed to overcome the energy barrier ($E_{a}$) of the reaction while the frequency factor ($A_{\mathrm{iso}}$) describes the rate of molecular collisions that results in a reaction. As shown in Fig. 1, $T_{p}^{\mathrm{thr}}$ is the same value in both the thermal profile and the τ versus $T_{p}$ square function. Therefore, correction of the isothermal to nonisothermal heating rate must come entirely from the frequency factor, which requires a definition different from the value represented by the y-intercept [Eq. (15)].

Values for $A_{\mathrm{non}}$ were determined for each threshold thermal profile, not the average thermal profiles, using Eq. (16) and the appropriate $E_{a}$ value from Table 1. The computed mean values of $A_{\mathrm{non}}$ and the standard deviations for each of the four exposure durations within each ambient temperature are listed in Table 2. Similar to how the antilog function causes large variances in $A_{\mathrm{iso}}$ data taken from the y-intercepts of Arrhenius plots, the exponential function of Eq. (16) causes large variances in values for $A_{\mathrm{non}}$. To illustrate this wide variance from similar data, we point out that the lines representing averaged thermal profiles in Fig. 2 include standard error of the mean (SEM) error bars (y-axis). Except for the 20-s exposures, the SEM error bars are negligible.

**Table 2:** Important parameters determined empirically or computationally. Data with STDEV values were derived using individual thermal profiles.

Target^a^ $T_{\mathrm{amb}}$ (°C)	Target^a^ τ (s)	$A_{\mathrm{non}}$ ± STDEV Eq. (16) ($s^{-1}$)	$A_{\mathrm{iso}}$ (Table 1) ($s^{-1}$)	$\Omega_{\mathrm{iso}}$ ± STDEV Eq. (8)	δ(τ) ± STDEV ($\Omega_{\mathrm{iso}}-1$) Eq. (11)	Threshold $T_{p}$ ± STDEV (empirical) (°C)	Threshold $T_{p}$ Eq. (17) (°C)
20			$3.09\times 10^{63}$				
	0.05	$1.47\pm 1.85\times 10^{65}$		0.27±0.33	−0.73±0.33	61.6±4.7	62.3
	0.25	$1.19\pm 0.46\times 10^{64}$		0.34±0.26	−0.66±0.26	59.1±1.2	58.6
	1	$4.85\pm 3.03\times 10^{63}$		0.95±0.61	−0.05±0.61	55.9±1.6	55.4^b^
	20	$8.89\pm 8.34\times 10^{63}$		1.08±1.72	0.08±1.72	48.7±2.6	48.8
25			$1.30\times 10^{65}$				
	0.05	$1.66\pm 1.18\times 10^{66}$		0.16±0.17	−0.84±0.17	62.7±2.0	63
	0.25	$6.07\pm 6.42\times 10^{65}$		0.36±0.19	−0.64±0.19	59.6±1.5	59.3
	1	$1.93\pm 0.89\times 10^{65}$		0.82±0.39	−0.18±0.39	56.4±1.1	56.3^b^
	20	$2.21\pm 1.27\times 10^{65}$		0.80±0.56	−0.20±0.56	50.0±1.4	49.8
30			$2.91\times 10^{82}$				
	0.05	$1.32\pm 2.19\times 10^{84}$		0.07±0.06	−0.93±0.06	59.5 ± 1.7	60.8
	0.25	$1.43\pm 1.42\times 10^{83}$		0.48±0.62	−0.52±0.62	58.2±1.7	58
	1	$1.80\pm 1.60\times 10^{83}$		0.38±0.34	−0.62±0.34	54.6±1.9	55.6^b^
	20	$4.99\pm 2.42\times 10^{82}$		0.75±0.42	−0.25±0.42	51.1±0.8	50.5
40			$3.26\times 10^{104}$				
	0.05	$5.72\pm 4.19\times 10^{105}$		0.11±0.10	−0.89±0.10	62.6±1.3	62.9
	0.25	$3.19\pm 4.06\times 10^{105}$		0.44±0.65	−0.56±0.65	60.0±1.2	60.6
	1	$4.57\pm 3.00\times 10^{105}$		0.18±0.26	−0.82±0.26	56.5±1.6	58.7^b^
	20	$2.72\pm 2.07\times 10^{104}$		1.40±0.80	0.4±0.80	50.4±1.3	54.6

a Exact values for $T_{\mathrm{amb}}$ and τ for each thermal profile used when calculating parameters using equations listed.

b Threshold $T_{p}$ at 1-s exposure time solves to the threshold critical temperature [see also Eq. (18)].

Notice that $A_{\mathrm{non}}$ values depend on both $T_{\mathrm{amb}}$ and exposure duration. As mentioned in the previous paragraph, the correction for nonisothermal heating must come from the $A_{\mathrm{non}}$ values, and the threshold thermal profiles provided this distinction. The general trend within each ambient temperature was for $A_{\mathrm{non}}$ to decrease with increasing laser exposure duration. This is because longer exposure times require a lower rate of collisions between molecules for the reaction to take place. As a result, less correction is needed to account for deviation from thermal profiles. The values for $A_{\mathrm{non}}$ increase with overall increases in $T_{\mathrm{amb}}$, in a similar manner and magnitude as the $A_{\mathrm{iso}}$ values (Table 1).

To calculate individual correction factors, the specific $A_{\mathrm{non}}$ values generated by each threshold thermal profile were used, along with the appropriate $E_{a}$ value, using Eq. (11). Again, we show the resulting $\delta_{c}(\tau )$ values as means and standard deviations in Table 2. The means and SEM values for the $\delta_{c}(\tau )$ values within the 0.05, 0.25, 1, and 20 s groups in Table 2 were −0.85±0.08, −0.58±0.21, −0.42±0.2, and 0.006±0.44, respectively. As expected, there was a strong dependence of $\delta_{c}(\tau )$ on laser exposure duration. It is interesting to note that for 20-s exposure durations, the $\delta_{c}(\tau )$ values fluctuated around the zero value. As expected, the size of the correction factor decreases with increase in exposure duration, and it reaches the value of zero at some measure of thermal steady state.

Referring to Eq. (14), the mathematical expression of the y-intercept (within parentheses) reveals a shift in the correction factor before taking the logarithm ($\mathrm{Ln}(\;A_{\mathrm{non}}(\delta_{c}(\tau )+1))$). This necessarily means that the $A_{\mathrm{non}}$ is always greater than or equal to the $A_{\mathrm{iso}}$ [Eq. (2)], for a negative value of the correction factor. As shown in Table 2, short laser exposure durations, such as those shorter than 1-s in our *in vitro* system, benefit greatly from correction of the damage integral with a nonisothermal frequency factor.

The fact that all the thermal profiles assessed were taken from the boundary of cell death, they meet the requirement for representing threshold events in a manner similar to an MVL.^7^ This is the basis for using these thermal profiles to calibrate the Arrhenius rate parameters. Slight variations in laser output, depth of buffer above the cells, cell cycle susceptibility, and the optical and thermal properties of the cell samples, all contributed to the temperature profile needed to cause cell death at the boundary of cell death. Thus, collectively the individual threshold thermal profiles provide a measure of heterogeneity, most of which was probably biological in nature. For this reason, we have chosen to use each thermal profile individually instead of the averaged thermal profiles. It is also the impetus for assigning a value of 1.0 for $\Omega_{\mathrm{non}}$ for the derivations and use of the nonisothermal equations (Sec. 2.1).

To compare with the $\Omega_{\mathrm{non}}=1$ values for all $T_{\mathrm{amb}}$ and τ combinations, we calculated $\Omega_{\mathrm{iso}}$ values using the ambient temperature dependent $E_{a}$ and $A_{\mathrm{iso}}$ values given in Table 1. This analysis is identical to the “Opt $E_{a}/A$” data in Table 3 of our prior publication,^7^ except here we are using the individual interpolated thermal profiles instead of the averaged thermal profiles. Table 2 provides the $\Omega_{\mathrm{iso}}$ values obtained from the “Opt $E_{a}/A$ method,” along with their differences from $\Omega_{\mathrm{non}}$ values. By dividing the $\Omega_{\mathrm{iso}}$ by $\Omega_{\mathrm{non}}$ (value of 1.0), the differences would be expressed as fractional differences. As shown in Table 2, for most combinations of exposure duration and $T_{\mathrm{amb}}$ the calculated $\Omega_{\mathrm{iso}}$ values are far less from the required value of 1.0 to confirm damage except for the 20-s exposure duration, where the steady state is considered an excellent approximation. Only the first 0.75 s of the 20-s exposure (Fig. 2) was not at, or near thermal steady state, which represents 3.75% of the exposure duration. This is the reason why $A_{\mathrm{iso}}$ is an excellent approximation for our 20-s exposures. Subsequently, the $\Omega_{\mathrm{iso}}$ calculated for the 20-s exposures was closer to a value of 1.0 than any other (Table 2).

**Table 3:** Linear dependence of the average heating rate on ambient temperature.

Laser exposure duration (s)	Linear equation^a^		
	y-intercept (°C $s^{-1}$)	Slope ($s^{-1}$)	Correlation ($R^{2}$)
0.05	1228.8	−19.9	0.98
0.25	230.7	−3.69	1.00
1	54.4	−0.96	0.99
20	1.9	−0.03	0.97

a Derived from data in Fig. 3(a) at constant exposure duration.

Without correcting the $A_{\mathrm{iso}}$ to $A_{\mathrm{non}}$ using $\delta_{c}(\tau )$ for the nonisothermal heating rates, most of the $\Omega_{\mathrm{iso}}$ values calculated from the known MVL thermal profiles would provide modelers erroneous indications for cell damage. Indeed, we concluded in our previous report that there was a propensity for underestimating cell death (Table 3 in Ref. 7). The correction factors given in Table 2 provide quick reference to the trends in erroneous damage estimates relative to $T_{\mathrm{amb}}$ and laser exposure duration.

#### 3.2.2 Threshold heating rate is reciprocally related to laser exposure duration

As indicated in previous sections, the frequency factor in a nonisothermal process ($A_{\mathrm{non}}$) changes as a function of temperature. Thus, it is not a constant parameter throughout the exposure duration, and it increases as a function of time and is expected to remain constant when the temperature reaches steady state. The values of $A_{\mathrm{non}}$ computed here were mean values for each exposure duration using threshold thermal profiles so they, along with the appropriate $E_{a}$ values, are suited for calculating damage rates (Arrhenius) and accumulated damage (nonisothermal damage integral) for the laser-induced damage processes in our cell culture model.

Rather than studying the complex relationships between $A_{\mathrm{non}}$ and both ambient temperature and laser exposure duration shown in Table 2, a natural extension is to examine the average heating rates ($\beta_{\mathrm{avg}}=\frac{1}{\tau }\int_{T_{\mathrm{amb}}}^{T_{p}^{\mathrm{thr}}}dT$). As expected, the heating rate was inversely proportional to both laser exposure duration and ambient temperature. Figure 3(a) shows that the heating rate needed to cause damage (threshold thermal profiles) depended greatly on $T_{\mathrm{amb}}$ and laser exposure duration. Average heating rates across $T_{\mathrm{amb}}$ (same τ value) were linear (lines not shown). Rather than providing equations for the $\beta_{\mathrm{ave}}$ versus $T_{\mathrm{amb}}$ in Fig. 3(a), we summarize the y intercepts and slopes, with correlation coefficients, in Table 3. Both Fig. 3(a) and Table 3 indicate that $\beta_{\mathrm{avg}}$ must necessarily be larger at lower ambient temperatures to obtain the τ-dependent peak temperature for threshold damage. Note that the dependence on $T_{\mathrm{amb}}$ (slopes, Table 3) was greatest for the shortest exposure durations.

**Fig. 3:**
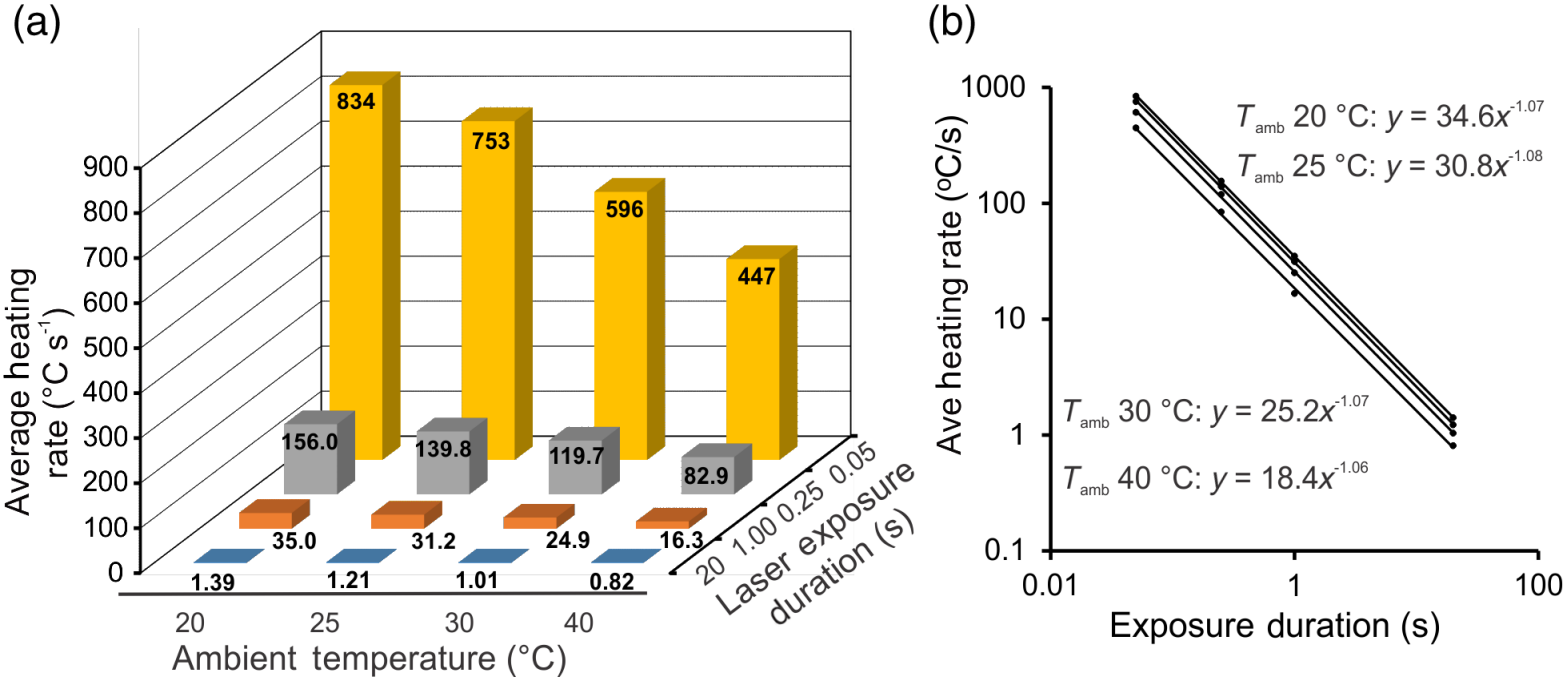
Threshold average heating rates in RPE cells exposed to 2-μm laser light. (a) Bar graph showing the relationship between both ambient temperature and laser exposure duration with average heating rate. Bars of yellow, gray, orange, and blue represent exposure durations of 0.05, 0.25, 1.0, and 20 s, respectively. (b) Power function trends between average heating rate and exposure duration. Slopes (exponents of power functions) indicate a reciprocal relationship ($\tau^{-1}$) between $\beta_{\mathrm{ave}}$ and τ. Note that $T_{\mathrm{amb}}$ + prefactor = $T_{\mathrm{crit}}^{\mathrm{thr}}$. All correlation coefficients ($R^{2}$) values were unity.

The average heating rates within the $T_{\mathrm{amb}}$ groups [Fig. 3(b)] had a similar power function relationship to laser exposure duration, where each slope (exponent) indicated that $\beta_{\mathrm{avg}}$ is not just inversely proportional but reciprocally related to laser exposure duration ($\beta_{\mathrm{avg}}\;\alpha$ $\tau^{-1}$). It is interesting to note that this reciprocal relationship extends to all four durations, regardless of whether or not the threshold thermal profile achieved thermal steady state [Fig. 3(b)].

The trends shown in Fig. 3(b) provide more information than just a reciprocal relationship. The prefix values for the power functions, by definition, are the values of $\beta_{\mathrm{avg}}$ at 1 s. When the ambient temperature (Table 1 mean value) was added to the $\beta_{\mathrm{avg}}$ at 1 s, we get our expected critical temperature, $T_{\mathrm{crit}}^{\mathrm{thr}}$ (threshold peak temperature at 1 s). In this way, the $T_{\mathrm{crit}}^{\mathrm{thr}}$ values derived from $\beta_{\mathrm{avg}}$ are 54.8°C, 55.6°C, 54.8°C, and 58.4°C for 20.2°C, 24.8°C, 29.6°C, and 40.0°C ambient temperature (Table 1), respectively. The $T_{\mathrm{crit}}^{\mathrm{thr}}$ values from our $\beta_{\mathrm{avg}}$ analysis from 20°C to 30°C $T_{\mathrm{amb}}$ were in line with the values (within the standard deviations) in Table 2 (peak temperature for 1-s exposures). The $T_{\mathrm{crit}}^{\mathrm{thr}}$ value of 58.4°C was only 0.3°C above the standard deviation range for the empirical value in Table 2. This result suggests that if one can empirically determine the $T_{\mathrm{crit}}^{\mathrm{thr}}$ value in any experimental system (cell culture, skin, retina) using threshold thermal profiles determined for 1-s exposures at a given $T_{\mathrm{amb}}$ (e.g., body temperature), one can determine the heating rate required for that 1-s exposure, as well as any other exposure durations by applying the reciprocal relationship to τ. More experimental data are needed to confirm this relationship.

#### 3.2.3 Nonisothermal predictive model

##### Threshold peak temperatures

Methods to compare the predictive consequences of using $A_{\mathrm{iso}}$ rather than $A_{\mathrm{non}}$ include comparing $\Omega_{\mathrm{non}}$ (e.g., 1.0) to $\Omega_{\mathrm{iso}}$ values calculated from either threshold thermal profiles or the values for $\delta_{c}(\tau )+1$. As shown in a previous section, the $A_{\mathrm{iso}}$ is a valid approximation under steady state conditions only. However, the $A_{\mathrm{non}}$ is valid for all exposure durations and is thus applicable to isothermal and nonisothermal heating equally well.

Another metric to examine the validity of correcting for non-isothermal heating rates is the calculation of threshold peak temperatures ($T_{p}^{\mathrm{thr}}$), which is analogous to the “$T_{\mathrm{TH}}$” value derived by Pearce and Thomsen.^19^ Table 2 compares differences between empirical threshold $T_{p}$ values relative to the $T_{p}^{\mathrm{thr}}$ values calculated using $A_{\mathrm{non}}$ in the following equation, derived from Eq. (14):

$$T_{p}^{\mathrm{thr}}=\frac{E_{a}}{R\;\mathrm{Ln}(\tau \cdot A_{\mathrm{non}}(\delta_{c}(\tau )+1))}.$$ (17)

Notice Eq. (17) depends upon the laser exposure duration. The $E_{a}$ value (energy requirement) drives the damage process to account for the $T_{\mathrm{amb}}$ by requiring the proper temperature rise. In addition, the $A_{\mathrm{non}}$ values influence $T_{p}^{\mathrm{thr}}$ values due to both $T_{\mathrm{amb}}$ and τ trends (Table 2). Similar dependencies are true for calculating the threshold critical temperature ($T_{\mathrm{crit}}^{\mathrm{thr}}$), which is Eq. (17) solved with an exposure duration of 1 s, similar to that derived by Pearce and Thomsen.^19^ Equation (18) provides the simplified expression for the critical temperature:

$$T_{\mathrm{crit}}^{\mathrm{thr}}=\frac{E_{a}}{R\;\mathrm{Ln}(A_{\mathrm{non}}(\delta_{c}(\tau )+1))}.$$ (18)

Notice how Eqs. (17) and (18) allowed the prediction of these two important threshold temperature values without a thermal profile. Once the proper threshold $E_{a}$ and $A_{\mathrm{non}}$ values are obtained, using a statistically significant number of threshold thermal profiles in a given system, $T_{p}^{\mathrm{thr}}$ and $T_{\mathrm{crit}}^{\mathrm{thr}}$ values can be predicted for any given laser exposure duration and ambient temperature for that system. At least this seems true in our *in vitro* retinal model.

The closer a damage rate process model can predict $T_{p}^{\mathrm{thr}}$ and $T_{\mathrm{crit}}^{\mathrm{thr}}$ values relative to empirical values, the more optimal the model. All the $T_{p}^{\mathrm{thr}}$ and $T_{\mathrm{crit}}^{\mathrm{thr}}$ (τ=1) reported in Table 2 fall within the standard deviations of the empirical threshold $T_{p}$ values, also shown in Table 2. This validates the predictive power of Eqs. (17) and (18) that uses $A_{\mathrm{non}}$ values. Most importantly, $A_{\mathrm{non}}$ not only accurately predicts the $T_{p}^{\mathrm{thr}}$ values, it maintains the required value of unity for $\Omega_{\mathrm{non}}$ to confirm damage. This is the great advantage of taking the additional work needed to get the $A_{\mathrm{non}}$ value.

##### Average heating rate

For processes carried out at a constant heating rate (β), $=\frac{dT}{dt}$, Eq. (5) can be reorganized to Eq. (19):

$$\frac{d\alpha }{dT}=f(\alpha )\frac{A_{\left(t^{\prime }\right)}}{\beta }e^{\frac{-E_{a}}{RT(t)}}.$$ (19)

Notice that assuming constant temperature (isothermal approach), β=0, generates an invalid assumption in the kinetic equation, eliminating its usage for analyzing nonisothermal laser-induced damage to tissue. The temperature history at the boundary location of cell death provides an estimate of the average heating rate required for threshold damage, ($\beta_{\mathrm{ave}}^{\mathrm{thr}}(\tau )$), as a function of exposure duration. As previously indicated, threshold thermal damage is reached by means of a peak temperature and a heating rate together. In a thermal process with a heating rate below threshold, reaching the threshold peak temperature is not likely to result in the anticipated damage. Calculations of the threshold average heating rate for each exposure duration and ambient temperature used all 198 thermal profiles collected at the boundary of the cell death were tabulated (data not shown). Using the empirical data, we established a two-dimensional (2D) fit of the heating rate $\beta (\tau ,T_{B})$ as a function of $T_{\mathrm{amb}}$ and τ at the boundary of the cell death was determined to be

$$\beta (\tau ,T_{\mathrm{amb}})=e^{-(\frac{\ln(\tau )+0.0284*T_{\mathrm{amb}}-3.8828}{0.93})}.$$ (20)

Equation (20) is an empirical formula, but it was not a simple “best-fit” analysis. One can convey the analysis as that being a method by which the complex relationships between $\beta_{\mathrm{ave}}^{\mathrm{thr}}$ and $T_{\mathrm{amb}}$ and τ, shown in Fig. 3, were iteratively related until the formula shown was found to consistently relate the two variables to the correct value for $\beta (\tau ,T_{\mathrm{amb}})$.

**Fig. 4:**
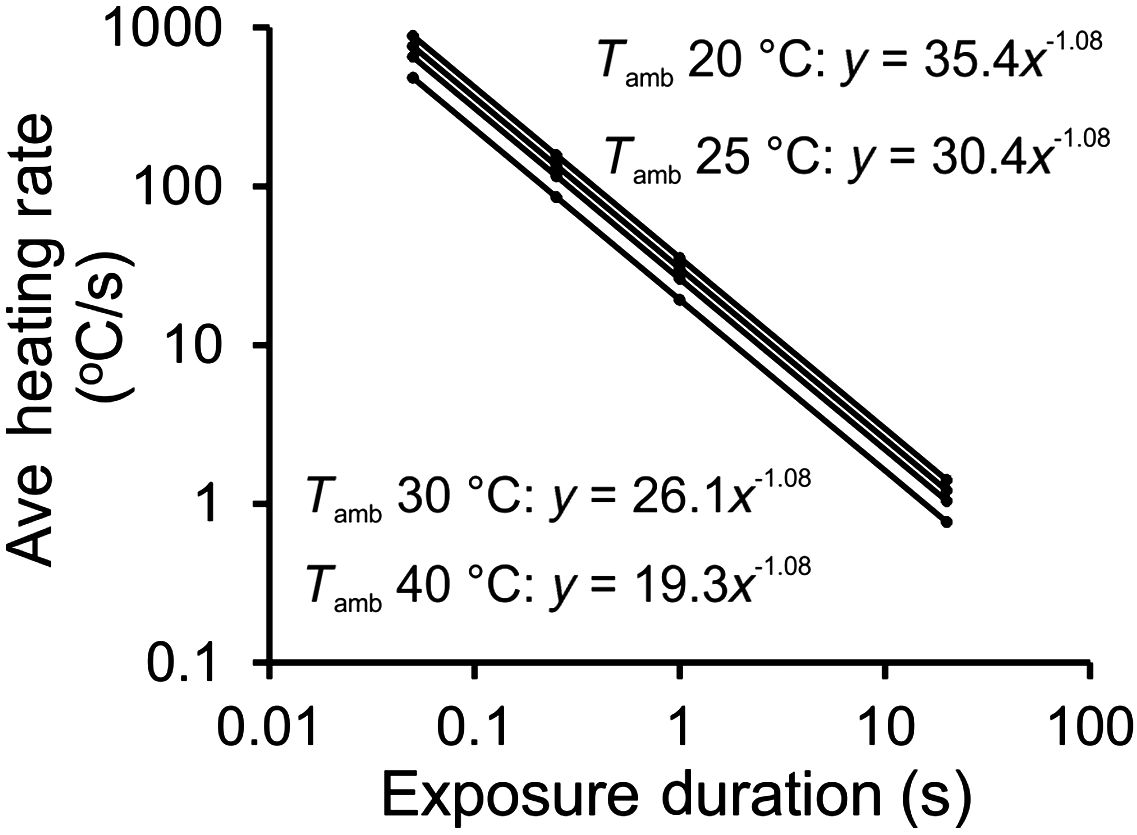
Plots of Eq. (20) at 20°C, 25°C, 30°C, and 40°C ambient temperature (solid lines). Calculated data points at 0.05, 0.25, 1.0, and 20 s are related to power functions (insets). The equations for the calculated values are essentially identical to those determined empirically [Fig. 3(b)]. Again, the power functions indicate reciprocity ($\tau^{-1}$) and the $T_{\mathrm{amb}}$ + prefactor = $T_{\mathrm{crit}}^{\mathrm{thr}}$. All correlation coefficients ($R^{2}$) values were unity.

Figure 4 shows the plots of Eq. (20) for varying laser exposure durations at the same four ambient temperatures shown in Fig. 3(b). Notice the similarities between Figs. 3(b) and 4, which indicates a robustness represented using the empirical data to formulate Eq. (20). When the data points for the 16 exposure types ($T_{\mathrm{amb}}$ and exposure durations) were used to obtain power functions in Microsoft Excel [as was done in Fig. 3(b)], we obtained similar equations to those in Fig. 3(b). The equations provided in Fig. 4 indicate the same reciprocal relationship and very similar $T_{\mathrm{crit}}^{\mathrm{thr}}$ values [Eq. (18)]. This agreement between the empirical heating rates and the best-fit 2D function validates the predictability of Eq. (20) in our cell culture system using only the $A_{\mathrm{non}}$ and $E_{a}$ values.

## 4 Summary and Conclusions

A nonisothermal form of the Arrhenius kinetic rate equation [Eq. (6)] was derived from a heterogeneous condensed phase system. By definition, use of threshold thermal profiles, like those from the boundary of cell death or the central temperature of a minimum visible lesion, to integrate the nonisothermal Arrhenius temperature integral [Eq. (7)] allows setting $\Omega_{\mathrm{non}}=1$. We determined the nonisothermal frequency factor ($A_{\mathrm{non}}$) for every threshold thermal profile (198) by rearranging $\Omega_{\mathrm{non}}$ to solve for $A_{\mathrm{non}}$ [Eq. (16)], which provided a statistical assessment. Once $A_{\mathrm{non}}$ was determined, the correction factor, which removes the overestimate identified in Fig. 1(a), can be determined using each threshold thermal profile as well. The correction factor is useful in identifying how disparate the $\Omega_{\mathrm{iso}}$ values would be if not corrected (Table 2).

The values for $\Omega_{\mathrm{iso}}$ reported here are not calculated by integrating the isothermal Arrhenius temperature integral, using the square function [Fig. 1(a)], which would lead to the worst-case scenario. Instead, like commonly done in the field of laser–tissue interaction, we integrated (Ω) thermal profiles using $A_{\mathrm{iso}}$ values obtained from Eq. (2) and Fig. 1(b), which averages across exposure durations. Results in Table 2 show the importance of determining $A_{\mathrm{non}}$ for the short, nonisothermal laser exposure durations. As noted in prior reports,^7^^,^^15^ our data underpin the notion that $\Omega_{\mathrm{iso}}$ values at exposure durations that do not lead to significant thermal steady state are greatly underestimated. We show that this underestimate is associated with $A_{\mathrm{iso}}$ values and the correction factor gives the degree of error.

This underestimate in damage accumulation (Ω) might be of interest to those assessing risk for damage using actual or simulated thermal profiles, taking note of the comparisons available in Table 2. We believe an order of magnitude underestimate for damage accumulation from 0.05-s exposures is significant. Existing data, which used the $A_{\mathrm{iso}}$ method, could now be corrected for nonisothermal temperature rise if desired. By returning to the original sample system and collecting a statistically significant number of threshold thermal profiles (e.g., MVL or boundary of cell death), one can calculate the $\delta_{c}(\tau )$, the $A_{\mathrm{non}}$, and set the $\Omega_{\mathrm{non}}$ value to unity. The difficulty in taking advantage of this correction is calibrating the sample system using threshold thermal profiles. The ability to measure and extract threshold thermal profiles, defined as having a value of $\Omega_{\mathrm{non}}=1$ as shown by our group,^7^^,^^15^ is demanding, but of paramount importance. These thermal profiles standardize the damage integral via the nonisothermal frequency factor.

In addition to its use in threshold damage assessments, the $\Omega_{\mathrm{non}}$ value, due to its accuracy, provides an estimate for varying degrees of damage. Currently, the ANSI Z136 standard uses a margin of approximately 10-fold between the empirical irradiance damage threshold in the nonhuman primate retina, and its minimum permissible exposure (MPE) value for safe use of lasers. Our results, based on thermal data rather than laser irradiance, indicate that a fractional value for $\Omega_{\mathrm{non}}$ might be used similarly, as a maximum permissible thermal exposure (MPTE). Without the variability in sample absorption, and being a thermal metric rather than a laser dose, the MPTE could be set with less margin than the MPE and still provide a safety buffer for potentially hazardous laser exposures. With current progress in thermal models based on physics first principles, simulation of thermal profiles from exposure to known laser parameters supports the use of the MPTE metric in the future if the method is found to be universal among physical models.

Table 2 also provides evidence that the $A_{\mathrm{non}}$ is more dependent upon temperature and exposure duration than previously assumed and reported.^19^ This, of course, leads to differences between the $\Omega_{\mathrm{iso}}$ values and the value of unity for $\Omega_{\mathrm{non}}$. The dependencies of $A_{\mathrm{non}}$ on $T_{\mathrm{amb}}$ and τ are complex. Threshold peak temperature ($T_{p}^{\mathrm{thr}}$) is also dependent upon both $T_{\mathrm{amb}}$ and τ, but without drastic swings in value, like $A_{\mathrm{non}}$. However, damage assessment cannot only use $T_{p}^{\mathrm{thr}}$ because the rate of heating plays a major role in damage as well (time-temperature history). Using our threshold thermal profiles, we determined that the average threshold heating rate ($\beta_{\mathrm{avg}}$) is linear across ambient temperature (Table 3) and reciprocally related across exposure duration [Fig. 3(b)]. This implies that if the exposure duration is doubled, the required threshold $\beta_{\mathrm{avg}}$ is cut in half. Based on these empirical results, we have derived a 2D empirical formula [Eq. (20)] that predicts the heating rate as a function of exposure duration and ambient temperature. We realize our results and assumptions are limited to our hTERT-RPE1 model. To assess for a broader applicability, we have begun to study different cell types in culture, and even extend to *in vivo* models, such as laser damage on skin.^22^

Finally, once the corrected A factor for nonisothermal heating is obtained, we can calculate both $T_{p}^{\mathrm{thr}}$ and $T_{\mathrm{crit}}^{\mathrm{thr}}$ in the same manner as shown by Pearce and Thomsen^19^ [Eqs. (17) and (18)]. The advantage to using the $A_{\mathrm{non}}$ values in these equations is that one can calculate the threshold temperature rises for the same system that correctly predicts $\Omega_{\mathrm{non}}=1$, and the precise threshold average heating rate. Combined, these parameters provide a more complete picture of the physical state required to cause threshold damage in our model system.

## References

[1] Henriques F. C. Moritz A. R., “Studies of thermal injury in the conduction of heat to and through skin and the temperatures attained therein: a theoretical and experimental investigation,” Am. J. Pathol. 23, 531–549 (1947). AJPAA4 0002-9440 PMC1934298 19970945

[r2] Moritz A. R. Henriques F. C., “Studies of thermal injury II. The relative importance of time and surface temperature in the causation of cutaneous burns,” Am. J. Pathol. 23, 695–720 (1947). AJPAA4 0002-9440 19970955 PMC1934304

[r3] Moritz A. R., “Studies of thermal injury III. The pathology and pathogenesis of cutaneous burns: an experimental study,” Am. J. Pathol. 23, 915–934 (1947). AJPAA4 0002-9440 19970971 PMC1934331

[4] Henriques F. C., “Studies of thermal injury V. The predictability and significance of thermally induced rate processes leading to irreversible epidermal injury,” Arch. Pathol. 43, 489–502 (1947). 20243514

[5] Arrhenius S. A., “About the speed of reaction in the inversion of cane sugar by acids,” Z. Phys. Chem. 4, 226–248 (1889). 10.1515/zpch-1889-0416

[6] Maiman T., “Optical and microwave-optical experiments in ruby,” Phys. Rev. Lett. 4, 564–566 (1960). PRLTAO 0031-9007 10.1103/PhysRevLett.4.564

[7] Denton M. L. et al., “Effect of ambient temperature and intracellular pigmentation on photothermal damage rate kinetics,” J. Biomed. Opt. 24(6), 065002 (2019). JBOPFO 1083-3668 10.1117/1.JBO.24.6.065002 31230427 PMC6977020

[8] Irvin L. et al., “BTEC thermal model,” USAF Technical Report AFRL-RH-BR-TR-2008-0006 (2008).

[9] Diller K. R. Pearce J. A., “Issues in modeling thermal alterations in tissues,” Ann. N.Y. Acad. Sci. 888, 153–164 (1999). ANYAA9 0077-8923 10.1111/j.1749-6632.1999.tb07954.x 10842631

[10] deWit J. N. Klarenbeek G., “Effects of various heat treatments on structure and solubility of whey proteins,” J. Dairy Sci. 67, 2701–2710 (1984). JDSCAE 0022-0302 10.3168/jds.S0022-0302(84)81628-8

[11] deWit J. N., “Nutritional and functional characteristics of whey proteins in food products,” J. Dairy Sci. 81, 597–608 (1998). JDSCAE 0022-0302 10.3168/jds.S0022-0302(98)75613-9 9565865

[12] Despa F. et al., “The relative thermal stability of tissue macromolecules and cellular structure in burn injury,” Burns 31(5), 568–577 (2005). BURND8 0305-4179 10.1016/j.burns.2005.01.015 15993302

[13] Vyazovkin S. Wight C. A., “Isothermal and non-isothermal kinetics of thermally stimulated reactions of solids,” Int. Rev. Phys. Chem. 17(3), 407–433 (1998). IRPCDL 0144-235X 10.1080/014423598230108

[14] Flynn J. H., “Thermal analysis kinetics-problems, pitfalls and how to deal with them,” J. Therm. Anal. 34(1), 367 (1988). 10.1007/BF01913405

[15] Denton M. L. et al., “Errata: spatially correlated microthermography maps threshold temperature in laser-induced damage,” J. Biomed. Opt. 20(7), 079801 (2015). JBOPFO 1083-3668 10.1117/1.JBO.20.7.079801 26222964

[r16] Koga N., “Ozawa’s kinetic method for analyzing thermoanalytical curves,” J. Therm. Anal. Calorim. 113, 1527–1541 (2013). JTACF7 1418-2874 10.1007/s10973-012-2882-5

[r17] Birngruber R., “Thermal modeling in biological tissues,” in Lasers in Biology and Medicine, Hillenkamp F. Pratesi R. Sacchi A., Eds., pp. 77–97, Plenum, New York (1980).

[r18] He X. Bischof J. C., “Quantification of temperature and injury response in thermal therapy and cryosurgery,” Crit. Rev. Biomed. Eng. 31(5&6), 355–422 (2003). CRBEDR 0278-940X 10.1615/CritRevBiomedEng.v31.i56.10 15139301

[r19] Pearce J. Thomsen S., “Rate process analysis of thermal damage,” in Optical Thermal Response of Laser-Irradiated Tissue, Welch A. J. van Germert M. J. C., Eds., 2nd ed., pp. 487–549, Plenum, New York (2011).

[r20] Jacques S. L., “Ratio of entropy to enthalpy in thermal transitions in biological tissues,” J. Biomed. Opt. 11(4), 041108 (2006). JBOPFO 1083-3668 10.1117/1.2343437 16965136

[21] Wright N. T. Humphrey J. D., “Denaturation of collagen via heating: an irreversible rate process,” Annu. Rev. Biomed. Eng. 4, 109–128 (2002). ARBEF7 1523-9829 10.1146/annurev.bioeng.4.101001.131546 12117753

[22] DeLisi M. P. et al., “Computational modeling and damage threshold prediction of continuous-wave and multiple-pulse porcine skin laser exposures at 1070 nm,” J. Laser Appl. 33, 022023 (2021). JLAPEN 1042-346X 10.2351/7.0000367

