# Damage integral and other predictive formulas for nonisothermal heating during laser exposure (Erratum)

**DOI:** 10.1117/1.JBO.31.5.059801

**Published:** 2026-05-29

**Authors:** Elharith M. Ahmed, Gary D. Noojin, Michael L. Denton

**Affiliations:** a SAIC, JBSA Fort Sam Houston, Texas, United States; b Air Force Research Laboratory, JBSA Fort Sam Houston, Texas, United States

## Abstract

The erratum provides details of a correction to the originally published article.

This article [*J. Biomed. Opt.* **27**(3), 035001 (2022) [doi 10.1117/1.JBO.27.3.035001] was originally published on 31 March 2022 with an error (typo) in Table 2.

Specifically, in the last row of Table 2 (40°C ambient temperature and 20-s exposure), the value indicated for the Threshold T_p_ (penultimate column) was incorrect:

• Original value: 56.4+/−1.3
• Corrected value: 50.4+/−1.3

Additionally, readers were mistakenly referred to Fig. 2 instead of Fig. 1 on page 035001-9. In Section 3.2.1 the following adjustment directs readers to the correct figure.

• Original text: “…in a reaction. As shown in Fig. 2, $T_{p}^{thr}$ is the same value in both the…”
• Corrected: “…in a reaction. As shown in Fig. 1, $T_{p}^{thr}$ is the same value in both the…”

The typos and corrections do not affect the scope or the conclusions of the published article. However, the article was corrected and republished 13 May 2026 to ensure technical accuracy.

